# Comparison of selected canine vector-borne diseases between urban animal shelter and rural hunting dogs in Korea

**DOI:** 10.1186/1756-3305-3-32

**Published:** 2010-04-08

**Authors:** Sun Lim, Peter J Irwin, SeungRyong Lee, MyungHwan Oh, KyuSung Ahn, BoYoung Myung, SungShik Shin

**Affiliations:** 1 Biotherapy Human Resources Center (BK21), Chonnam National University, Gwangju 500-757, Korea; 2 Australasian Centre for Companion Animal Research, School of Veterinary and Biomedical Sciences, Murdoch University, Murdoch 6150, WA, Australia; 3 College of Veterinary Medicine, Chonnam National University, Gwangju 500-757, Korea; 4 Gwangju Animal Shelter, Gwangju 500-757, Korea

## Abstract

A serological survey for *Dirofilaria immitis*, *Anaplasma phagocytophilum*, *Ehrlichia canis*, and *Borrelia burgdorferi *infections in rural hunting and urban shelter dogs mainly from southwestern regions of the Republic of Korea (South Korea) was conducted. From a total of 229 wild boar or pheasant hunting dogs, the number of serologically positive dogs for any of the four pathogens was 93 (40.6%). The highest prevalence observed was *D. immitis *(22.3%), followed by *A. phagocytophilum *(18.8%), *E. canis *(6.1%) and the lowest prevalence was *B. burgdorferi *(2.2%). In contrast, stray dogs found within the city limits of Gwangju showed seropositivity only to *D. immitis *(14.6%), and none of the 692 dogs responded positive for *A. phagocytophilum*, *E. canis *or *B. burgdorferi *antibodies. This study indicates that the risk of exposure to vector-borne diseases in rural hunting dogs can be quite high in Korea, while the urban environment may not be suitable for tick infestation on dogs, as evidenced by the low infection status of tick-borne pathogens in stray dogs.

## Findings

The situation with respect to parasitic diseases of companion animals in the Republic of Korea (South Korea) still remains relatively uninvestigated. Especially, limited information is available on the status of vector-borne disease transmission among dogs and cats. As global warming is affecting climate conditions of Korea, subtropical parasitic diseases such as malaria that has not been established in South Korea are now emerging [1].

Canine vector-borne pathogens which include *Dirofilaria *spp., *Anaplasma *spp., *Ehrlichia *spp., *Borrelia *spp. and others can elicit serious illness in domestic dogs. These agents can also cause clinical illness such as human dirofilariasis as a result of accidental infection [2]. Lyme disease, anaplasmosis, and infections with *Ehrlichia canis *have been reported in humans, too [3-5]. Canine vector-borne diseases have been found throughout major continents of the world [6,7]. In Japan, the prevalence of *E. canis *was 4.7% [8] while that of *B. burgdorferi *was 8.8% in dogs [9]. In Korea, little information is available regarding the occurrence of these diseases in dogs, although the prevalence in ticks and small mammals has been well documented [10,11]. With regard to the dog, most studies on vector-borne diseases have focused on canine heartworm disease, which has a prevalence ranging from 9.9% to 50.3% [12-16]. Since outdoor dogs such as hunting, military or stray dogs are vulnerable to vector-borne pathogens, we investigated the prevalence of *D. immitis*, *A. phagocytophilum*, *E. canis*, and *B. burgdorferi *among hunting and stray dogs from Korea.

From December of 2007 to August of 2009, blood samples were collected from 229 hunting dogs in Beolgyo, Gwangyang, Suncheon, and Asan areas of South Korea. These areas are located from 34° 50' N to 35° 05' N latitude and from 127° 15' E to 127° 34' E longitude (southwestern region of South Korea) except for Asan which is located 36° 45' N latitude and 126° 89' E longitude (mid-western region of South Korea). Dogs included in this study were raised for the purpose of hunting either pheasants or wild boars with an average of 3.2 years of age and an average body weight of 23.3 Kg. The majority of dogs were cross breeds of German Shorthaired Pointer, and were composed of 145 (63.3%) male and 84 (36.7%) female dogs. Blood samples were also collected from a total of 692 stray dogs admitted to the Gwangju Animal Shelter from January to December of 2009. The city of Gwangju, with a population of 1.4 million people in December 2009, is also located in the southwestern region of Korea where Beolgyo, Gwangyang and Suncheon is situated within the distance of 100 Km from the city. The majority of stray dogs admitted to the only shelter of the city were either small- or middle-sized breeds with an average body weight of 4.0 Kg. Among them, Maltese (27.0%), mixed breeds (21.4%), Shih Tzu (16.0%), Yorkshire terrier (11.0%), and Poodle (7.5%) were the most commonly found breeds. Blood samples collected from dogs were tested using a commercial ELISA assay kit (SNAP^® ^4Dx^®^; IDEXX Laboratories, Inc. U.S.A.) which detects *D. immitis *antigen, and antibodies specific to *A. phagocytophilum *(synthetic peptide from the major surface protein (p44/MSP2)), *E. canis *(P30 and P30-1 outer membrane proteins), and *B. burgdorferi *(C6 peptide). A test of independence for significance of the relationship between categorical variables (gender, age, and geographic regions) was made via Pearson's chi-Square test and Fisher's exact test for expected counts under five using SPSS 17.0 (SPSS Inc., Chicago, IL, USA).

The serological prevalence of *D. immitis*, *A. phagocytophilum*, *E. canis*, and *B. burgdorferi *in hunting dogs from Korea is shown in Table 1. The number of dogs serologically positive with any of the four pathogens surveyed in this study was 93 (40.6%). The number of dogs with single, dual or triple seropositivity was 75 (32.8%), 16 (7.0%), and 2 (0.9%), respectively. The highest prevalence was observed in *D. immitis *(22.3%), followed by *A. phagocytophilum *(18.8%), and the lowest by *B. burgdorferi *(2.2%). Although a significant variation in geographical origin was observed in *E. canis *Ab (χ^2 ^= 7.968, *p *= 0.032: Fisher's exact test), the overall exposure of dogs to these pathogens was irrelevant to geographical locality (χ^2 ^= 0.848, *p *= 0.838). The number of serologically positive dogs was similar between male (41.4%) and female (39.3%, χ^2 ^= 0.097, *p *= 0.756), but dogs of above two years in age (45.3%) were significantly more exposed to these pathogens than younger dogs (24.0%, χ^2 ^= 7.318, *p *= 0.007) which was mostly influenced by the exposure of the dogs to *D. immitis *(χ^2 ^= 7.525, *p *= 0.006).

**Table 1:** Seroprevalence of selected arthropod-borne pathogens in hunting dogs from Korea as detected by a commercial screening test

		Dogs examined (%)	Number(%) of positive dogs by SNAP 4Dx test				
			
Category			Di Ag	Ap Ab	Ec Ab	Bb Ab	Total*
Gender	Female	84(36.7)	23(27.4)	13(15.5)	3(3.6)	2(2.4)	33(39.3)
	Male	145(63.3)	28(19.3)	30(20.7)	11(7.6)	3(2.1)	60(41.4)

Age(year)	<2	50(21.8)	4(8.0)	5(10.0)	3(6.0)	0(0.0)	12(24.0)
	>= 2	179(78.2)	47(26.3)	38(21.2)	11(6.1)	5(2.8)	81(45.3)

Geographical origin	Beolgyo	32(14.0)	7(21.9)	6(18.8)	4(12.5)	2(6.3)	14(43.8)
	Gwangyang	65(28.4)	18(27.7)	7(10.8)	3(4.6)	0(0.0)	27(41.5)
	Suncheon	26(11.4)	2(7.7)	7(26.9)	4(15.4)	1(3.8)	12(46.2)
	Asan	106(46.3)	24(22.6)	23(21.7)	3(2.8)	2(1.9)	40(37.7)

Total		229(100.0)	51(22.3)	43(18.8)	14(6.1)	5(2.2)	93(40.6)

*Number of positive dogs by any of the four test results by SNAP 4Dx test

Di Ag, *Dirofilaria immitis *antigen; Ap Ab, *Anaplasma phagocytophilum *antibody; Ec Ab, *Ehrlichia canis *antibody; Bb Ab, *Borrelia burgdorferi *antibody

The seroprevalence of selected arthropod-borne pathogens in stray dogs admitted to the Gwangju Animal Shelter during the year 2009 is shown in Table 2. Unlike the hunting dogs raised and living outside of the city, stray dogs found within the city limit of Gwangju showed seropositivity only to *D. immitis *(14.6%) and none of the 692 dogs responded positive for *A. phagocytophilum*, *E. canis *or *B. burgdorferi *antibodies. The number of serologically positive dogs was significantly more in female (17.9%) than in male (12.4%, χ^2 ^= 4.014, *p *= 0.045), and dogs of more than two years old were significantly more exposed to these pathogens than younger dogs (χ^2 ^= 7.611, *p *= 0.006).

**Table 2:** Seroprevalence of selected arthropod-borne pathogens in stray dogs admitted to a shelter in Gwangju, Korea as detected by a commercial screening test

		Dogs examined (%)	Number(%) of positive dogs by SNAP 4Dx test				
			
Category			Di Ag	Ap Ab	Ec Ab	Bb Ab	Total*
Gender	Female	280(40.5)	50(17.9)	0(0.0)	0(0.0)	0(0.0)	50(17.9)
	Male	412(59.5)	51(12.4)	0(0.0)	0(0.0)	0(0.0)	51(12.4)

Age(yr)	<2	211(30.5)	19(9.0)	0(0.0)	0(0.0)	0(0.0)	19(9.0)
	>= 2	481(69.5)	82(17.0)	0(0.0)	0(0.0)	0(0.0)	82(17.0)

Geographical origin (district)	East	58(8.4)	12(20.7)	0(0.0)	0(0.0)	0(0.0)	12(20.7)
	West	105(15.2)	15(14.3)	0(0.0)	0(0.0)	0(0.0)	15(14.3)
	South	87(12.6)	13(14.9)	0(0.0)	0(0.0)	0(0.0)	13(14.9)
	North	355(51.3)	53(14.9)	0(0.0)	0(0.0)	0(0.0)	53(14.9)
	Gwangsan	87(12.6)	8(9.2)	0(0.0)	0(0.0)	0(0.0)	8(9.2)

Total		692(100.0)	101(14.6)	0(0.0)	0(0.0)	0(0.0)	101(14.6)

*Number of positive dogs by any of the four test results by SNAP 4Dx test

Di Ag, *Dirofilaria immitis *antigen; Ap Ab, *Anaplasma phagocytophilum *antibody; Ec Ab, *Ehrlichia canis *antibody; Bb Ab, *Borrelia burgdorferi *antibody

This study strongly indicates that dogs from Korea are potentially vulnerable to exposure to major canine vector-borne diseases, as evidenced by the relatively high prevalence rates of both mosquito- and tick-borne pathogens in hunting dogs. Previous reports also indicate that vector-borne pathogens such as *E. chaffeensis*, *A. phagocytophilum*, and *A. bovis *were identified by TaqMan real-time PCR [17] from ticks collected from various areas of Korea. Also, five species of ticks in two genera (*Haemaphysalis *spp. and *Ixodes *spp.) collected from small wild-caught mammals or by dragging/flagging in Korea contained species-specific fragments of *A. phagocytophilum*, *A. platys*, *E. chaffeensis, E. ewingii, E. canis*, and *Rickettsia rickettsii*, as evidenced by the PCR assay [10].

While infection status of the mosquito-transmitted *D. immitis *infection was relatively high in both hunting and stray dogs, the tick-borne pathogens were present only in hunting dogs. Two factors may be involved to explain the result. First, although 38.9% of the 501 million m2 land of the city of Gwangju is covered with woods and fields, it is presumed that wild animals that can transmit ticks to dogs are rarely able to enter or persist in the urban environment. Secondly, the floor of people's homes has a special place in the culture of Koreans; it is generally polished and un-carpeted, on which they sit and often sleep. People always remove their shoes when entering a Korean home because a dirty floor is seldom tolerated in a Korean home. As the result, ticks and fleas are rarely found infesting urban indoor dogs of Korea. For the same reason, small dogs like Maltese, Yorkshire, and Shi Tzu are commonly preferred by pet owners in Korea because they are well adapted to being apartment dwellers. Stray dogs admitted to the Gwangju Animal Shelter very much represent dog breeds favoured by urban-dwelling Koreans; Maltese, Shih Tzu, Yorkshire terrier, Poodle, and Schnauzer, etc [18].

While mosquitoes are ever-present in the city environment and even indoor-only dogs can get bitten by them, this study indicates that ticks, in contrast, may have limited access to the city environment of Korea. Similar results were observed in 2008 from a previous study on the infection status of stray dogs at the same animal shelter as investigated in this study in which 130 of 1,143 stray dogs (11.4%) showed positive reaction to *D. immitis *on SNAP^® ^3Dx^® ^test, while only one dog each showed seropositive to *E. canis *and *B. burgdorferi*, respectively [18].

Since the first report of *D. immitis *in dogs from Korea was published in 1962 [19], there have been several studies on the epidemiology of canine dirofilariasis in Korea. The prevalence of *D. immitis *for instance, was 31.2% using an antigen test (Heartworm SNAP^® ^test, IDEXX, Inc.) in outdoor dogs and 2.8% in indoor dogs from Busan, Korea [12], and that in German shepherd using and antigen test (DiroCHEK^®^, Synbiotics Co., USA) was 28.3% [13]. In our studies, the prevalence of *D. immitis *in both hunting and stray dogs was similar to those of previous studies on outdoor dogs. In contrast to relatively low prevalence rate in dogs from the USA (1.4%) [20], the prevalence of *D. immitis *in dogs from Korea was high in general, possibly because of better public apprehension and prophylactic programs carried in the USA than in Korea.

Little information is available about the infection status of dogs with *A. phagocytophilum *which is also responsible for human granulocytic anaplasmosis [21]. Although the prevalence of *A. phagocytophilum *in ticks collected from small mammals at U.S. military installations and training sites was 25.9% as identified by DNA analysis in Korea [10], only one clinical case due to *A. platys *has so far been reported in dogs [22], and our study is the first report about seroprevalence of *A. phagocytophilum *in dogs from Korea. In the USA, the mean prevalence of *A. phagocytophilum *seroreactivity in dogs was reported to be 4.8% by SNAP^®^4Dx^® ^test [20]. In contrast to previous studies, hunting dogs in our study show a high prevalence of *A. phagocytophilum *seroreactivity (18.8%), presumably due to frequent exposure of dogs to vector ticks during hunting in wooded mountains of Korea. Information on the species of ticks collected from hunting dogs in our study will be available in a separate article. It is possible that some dogs with seroreactivity to *A. phagocytophilum *were actually seropositive for *A. platys *because both *A. phagocytophilum *and *A. platys *exist among ticks in Korea [10] and because the SNAP^®^4Dx^® ^cannot distinguish infection between *A. phagocytophilum *and *A. platys *in dogs. Further molecular-based studies will be necessary to distinguish between these two pathogens in seropositive dogs.

The prevalence of *E. canis *in ticks of Korea was 1.1%, as identified by DNA analysis [10], and the seroprevalence of *E. canis *in dogs (German shepherd) using the IDEXX^® ^3Dx^® ^test was reported to be 13% in female and 11.6% in male [23]. Both *E. canis *and *E. chaffeensis *are present in Korea, as detected from ticks [10]. Since SNAP^® ^3Dx^® ^and 4Dx^® ^tests are known not to be able to distinguish between *E. canis *and *E. chaffeensis *infections in dogs [24], it will be necessary to distinguish them by further investigation.

*B. burgdorferi *is a zoonotic pathogen because it causes Lyme disease in humans and infects some domestic mammals including dogs. In Korea, the seroprevalence of *B. burgdorferi *was reported to be 2.6% in female and 5.8% in male German shepherd dogs [23] and more than 4 clinical human cases have been reported [25]. *B. burgdorferi *was also isolated from ticks in 1992 [26]. The seroprevalence rate of *B. burgdorferi *in dogs was 1.3% in U.S [20] and 0.6% in Spain [27]. In our studies, the prevalence of *B. burgdorferi *in hunting dogs (2.2%) was similar to that of a previous study in German shepherd dogs in Korea [23].

In conclusion, this study indicates that hunting dogs are frequently exposed to *D. immitis*, *A. phagocytophilum*, *E. canis*, and *B. burgdorferi *in Korea while urban stray dogs are exposed mainly to *D. immitis*. Since canine vector-borne diseases can cause severe clinical illness such as pulmonary disease, lameness, fever and anorexia and can also potentially cause severe diseases in humans, dogs must be examined for the presence of vector-borne diseases.

## Competing interests

The authors declare that they have no competing interests.

## Authors' contributions

SL, PJI, SRL and SSS conceived the paper and wrote the manuscript. MHO, KSA, and BYM assisted in laboratory studies.
